# Influence of pacifier use on the association between duration of breastfeeding and anterior open bite in primary dentition

**DOI:** 10.1186/s12884-020-03054-z

**Published:** 2020-07-08

**Authors:** Vanessa Felipe de Deus, Erissandra Gomes, Fernanda Caramez da Silva, Elsa Regina Justo Giugliani

**Affiliations:** 1 Postgraduate Program in Child and Adolescent Health, Faculty of Medicine, Universidade Federal do Rio Grande do Sul (UFRGS), Rua Ramiro Barcelos, 2400, Porto Alegre, RS CEP 90035-003 Brazil; 2 Department of Surgery and Orthopedics, School of Dentistry, Universidade Federal do Rio Grande do Sul (UFRGS), Rua Ramiro Barcelos, 2492, Porto Alegre, RS CEP 90035-003 Brazil

**Keywords:** Breastfeeding, Anterior open bite, Dental occlusion

## Abstract

**Background:**

The literature is controversial with regard to the association between longer breastfeeding duration and lower prevalence of anterior open bite. Pacifier use may be involved in this controversy. Thus, the objective of the study was to assess the influence of pacifier use and its duration on the association between longer breastfeeding duration and lower prevalence of anterior open bite in children with primary dentition.

**Methods:**

This was a cross-sectional study nested in a cohort study involving 153 infants recruited at a maternity hospital in the municipality of Porto Alegre, southern Brazil. The study outcome (anterior open bite) was assessed when the children were between 3 and 5 years old. Data on breastfeeding and pacifier use were collected at 7, 30, 60, 120, and 180 days of life and on the date of the evaluation here described. Poisson regression with robust variance was used to analyze the association between the prevalence of anterior open bite and breastfeeding duration, expressed in months.

**Results:**

The univariate analysis showed a protective effect of breastfeeding against anterior open bite (prevalence ratio [PR] 0.96; 95% confidence interval [95%CI] 0.95–0.98). This effect remained significant after adjustment for pacifier use at any time between birth and the date of dental assessment (PR0.98; 95%CI; 0.96–0.99), i.e., there was a reduction of 2% in the prevalence of anterior open bite for each month of breastfeeding. However, this effect lost significance when pacifier use duration was included in the multivariate analysis (PR1.00; 95%CI; 0.99–1.01).

**Conclusions:**

Pacifier use duration influences the association between longer breastfeeding duration and lower prevalence of anterior open bite. It is likely that prolonged pacifier use reduces the magnitude of this association.

## Background

Anterior open bite (AOB) is a common dental malocclusion, especially in preschool children, characterized by a deficiency in normal vertical contact between the incisal edges of the upper and lower anterior teeth [1–3]. Depending on its extent, this malocclusion can impair mastication, swallowing and breathing, with a consequent decrease in quality of life; also, it can have a negative economic impact on the family, since its treatment is expensive [1, 4, 5]. Of multifactorial etiology, it is often linked to an orofacial myofunctional disorder, either due to genetic factors or due to the prolonged action of parafunctional habits, such as finger sucking and prolonged use of a pacifier [1, 2, 6, 7].

The hypothetical protective effect of breastfeeding on AOB can be explained by the greater activity of facial muscles during breast suction, which promotes adequate craniofacial growth, development of the mandible muscles and dental occlusion [4, 7, 8]. The association between longer duration of breastfeeding and lower prevalence of AOB has been confirmed by two recent meta-analysis [4, 5]. However, not all studies have confirmed this finding [9]. The discrepancy observed is due, at least in part, to methodological differences among the studies, the way the variable breastfeeding is expressed in terms of occurrence and duration, the type of dentition considered, as well as the age of the study subjects. Another factor that can interfere in the results of the studies are the deleterious oral habits, especially nonnutritive sucking (e.g. pacifier use), which are not always taken into account.

Using a pacifier has been associated with occlusal alterations [10–12] due to the hyperfunction of the buccinator muscle, causing restriction in the transverse growth of the mandible; and the low position of the tongue, preventing the necessary pressure against the hard palate and, consequently, the adequate growth and transversal development of the maxillary arch [1, 8, 13, 14].

The studies of breastfeeding duration and prevalence of AOB using multivariate analysis to adjust for the pacifier use variable show conflicting results [1, 2, 6, 7, 15]. Of these studies, two showed an association between longer breastfeeding duration and lower prevalence of AOB [1, 15], two showed association only in the univariate analysis, losing significance after adjustment [2, 6] and one showed no significant association at any point in the analysis [7].

This study was designed having in mind the divergences among studies regarding the protective effect of breastfeeding against development of AOB; the fact that not all studies consider pacifier use in their analysis; and that there are no studies that have investigated the association between breastfeeding duration and the prevalence of AOB considering the actual duration of breastfeeding and of pacifier use. Its purpose was to generate new information that is useful to expand knowledge in this area.

## Methods

### Study design

This was a cross-sectional study nested in a cohort study with the objective of evaluating the influence of pacifier use and its duration on the association between longer breastfeeding duration and lower prevalence of AOB in children with primary dentition using a novel approach, namely, considering breastfeeding duration and pacifier use duration as continuous rather than categorical variables. The cohort was originally designed to identify factors associated with interruption of exclusive breastfeeding in the first 6 months of life [16].

The sample size calculation for the cohort study was estimated considering the following parameters: power of 80, 95% confidence level, relative risk of 1.3, and exclusive breastfeeding prevalence among children under 6 months, unexposed to the different risk factors, of 30%. The minimum number of participants varied from 128 to 210, depending on the prevalence of exposure to the risk factors being studied (20–70%).

### Participants

Sampling was selected in a general university hospital in Porto Alegre, southern Brazil. Every day two mother-child pairs were randomly included in the study. Each eligible woman received a number for the drawing lot performed by one of the researchers. The limitation on the number of participants selected per day was due to logistical issues, as this is a prospective study, with frequent contacts during follow-up. To participate in the study, the neonates had to meet the following inclusion criteria: singletons, weighing ≥2500 g, rooming in, having started breastfeeding and whose family resided in the municipality where the study was conducted.

**Fig. 1:**
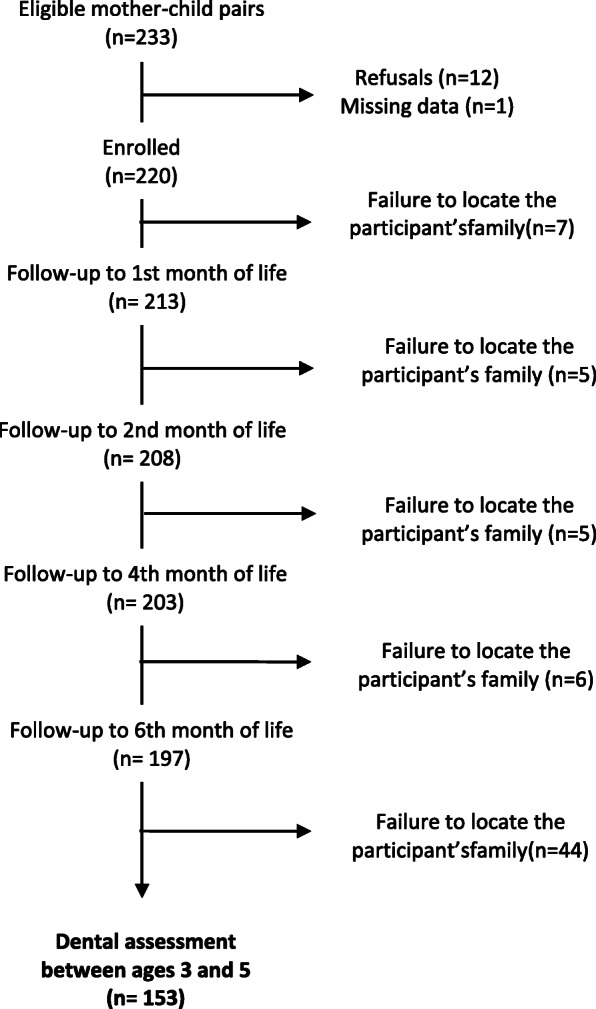
Cohort follow-up

Children included in the first phase of the study (*n* = 220) who were followed for the first 6 months of life (*n* = 197) were seen again at the age of 3–5 years (*n* = 153) and assessed for the occurrence of malocclusions, including AOB (Fig. 1). The sample was selected between June and November 2003.

The study was approved by the research ethics committee of Hospital de Clínicas de Porto Alegre under protocol no. 06–554/2007. All participating mothers signed an informed consent form.

### Data collection

Sociodemographic data were obtained via interviews conducted with the mothers of the infants selected, at the maternity ward, between the second and third day of life of the newborns. Data on feeding and sucking habits were obtained at 7 and 30 days of life via home visits, at 60, 120, and 180 days via telephone calls, and when the children were aged between 3 and 5 years. Thus, the data on breastfeeding duration was collected prospectively until the children were 6 months of age and retrospectively between this age and the last assessment.

The dental evaluation was carried out in the last contact when the children were 3–5 years old in the dental office or in their homes when the family did not attend the dental office. This evaluation was done by a senior dentist trained for the purposes of the study with the child seated and the teeth in maximum intercuspation position, i.e., with the maxillary and mandibular dental arches in contact, in the usual position for each patient. Only after completion of the clinical examination, data on feeding and nonnutritive sucking habits were collected covering the period ranging from 6 months of life until the date of the current examination, so as to avoid that previous knowledge of these data could influence clinical judgment.

AOB, the outcome of this study, was defined as the absence of vertical overlap between maxillary and mandibular incisors where the front teeth do not touch when the back teeth are closed together [3].

Breastfeeding duration was expressed as number of months elapsed from the infant’s birth to the termination of breastfeeding, regardless of the introduction of any other liquid or solid food in this period, including non-human milk. Pacifier use duration was expressed as number of months elapsed between the beginning and end of the use of this artifact. Whenever the child was still using a pacifier at the time of evaluation, the current age of the child was considered as the date of termination of pacifier use.

### Statistical analysis

Statistical analysis was performed using the Statistical Package for Social Science (SPSS) version 21.0 for Windows. Quantitative variables were expressed as mean and standard deviation or as median and interquartile range. Categorical variables were described as absolute and relative frequencies. For the comparison of median the Mann-Whitney test was used.

The association between prevalence of AOB and breastfeeding duration was tested initially through univariate analysis, followed by multivariate analysis using Poisson regression with robust variance. Sociodemographic variables that showed a significant association (p < 0.2) with the presence of AOB would enter the multivariable model, as well as the child age, to account for the wide age range of the children (3 to 5 years). As a result, two multivariate analyses were conducted, one considering only if the child used or did not use a pacifier at any time between birth and the date of evaluation (categorical variable), and another considering pacifier use duration in months (continuous variable).

## Results

The majority of children’s mothers was white and had completed elementary education; mean age was 24 years. Almost half of the children (44%) presented AOB (95% confidence interval [95%CI] 36.7–52.4) and approximately ¾ had used a pacifier at some point. Median duration of breastfeeding was a little below 1 year, and 32% of the children were breastfed for 2 years or more; only 5.9% were exclusively breastfed for the first 6 months (Table 1). Losses to follow-up were due to failure to locate the participant’s family (Fig. 1).

**Table 1:** Sociodemographic data, breastfeeding duration, and nonnutritive sucking habits

Variable	***n*** = 153
Mother age (years), mean ± SD	24.4 ± 6.3
Mother race white, *n* (%)	100 (65.4)
Schooling ≥8 years, *n* (%)	99 (64.7)
Primiparity, *n* (%)	78 (51.0)
Child age (months), mean ± SD	50.2 ± 7.2
Child sex male, *n* (%)	83 (54.2)
Breastfeeding duration (months), median (P25-P75)	11.5 (4–30)
Pacifier use at any time, *n* (%)	114 (74.5)
Pacifier use duration (months), median (P25-P75)	38.5 (0–45)
Anterior open bite, *n* (%)	68 (44.4)

*P25-P75* 25th–75th percentile, *SD* standard deviation

There was a statistically significant difference in the median duration of breastfeeding between children without and with AOB: median of 21 months, 25th–75th percentile (P25-P75) = 7.5–35 among children without AOB, vs. median of 5.3 months, P25-P75 = 2.2–13 among those with AOB. With regard to pacifier use duration, children with AOB showed a median of 43 months (P25-P75 = 40–50), differently from those who did not show the malocclusion, in which the median was 0 month (P25-P75 = 0–18) (*p* < 0.001).

No sociodemographic variable tested showed an association (*p* < 0.2) with the prevalence of AOB as follows: maternal age (*p* = 0,919); maternal schooling (*p* = 0,610); self-declared maternal race (*p* = 0,747); parity (*p* = 0,957); and child’s sex (*p* = 0,268).

Table 2 shows the results of the univariate and multivariate analyses in which pacifier use was categorized into yes (used a pacifier any time between birth and the dental evaluation) or no. Significant associations were observed in both analyses. After adjustment for age and pacifier use, a reduction of 2% was estimated in the prevalence of AOB for each month of breastfeeding. Children who used a pacifier at any time showed a 8-fold higher prevalence of AOB.

**Table 2:** Univariate and multivariate analyses conducted to test the association between presence of anterior open bite and breastfeeding duration, considering the use of a pacifier at any time

Variables	Crude PR (95%CI)	Adjusted PR (95%CI)	***p***-value
Breastfeeding duration (months)	0.96 (0.95–0.98)	0.98 (0.96–0.99)	0.007
Pacifier use	7.41 (2.47–22.2)	8.18 (2.05–32.7)	0.003
Child age (months)	0.98 (0.95–1)	0.98 (0.96–1)	0.047

*95%CI* 95% confidence interval, *PR* prevalence ratio

When pacifier use duration (in months) was considered in the multivariate analysis, the protective effect of breastfeeding against development of AOB lost significance. On the other hand, for each month of pacifier use, the prevalence of AOB increased by 5% (Table 3).

**Table 3:** Univariate and multivariate analyses conducted to test the association between presence of anterior open bite and breastfeeding duration, considering pacifier use duration

Variables	Crude PR (95%CI)	Adjusted PR (95%CI)	***p***-value
Breastfeeding duration (months)	0.96 (0.95–0.98)	1 (0.99–1.01)	0.992
Pacifier use (months)	1.04 (1.03–1.05)	1.05 (1.03–1.06)	< 0.001
Child age (months)	0.98 (0.95–1)	0.96 (0.94–0.99)	0.001

*95%CI* 95% confidence interval, *PR* prevalence ratio

## Discussion

The present study showed that pacifier use might influence the association between longer breastfeeding duration and lower prevalence of AOB in children with primary dentition. This association remained significant after the inclusion of pacifier use in the statistical model that disregarded its duration. Nevertheless, significance was lost when pacifier use duration was considered in the analysis. A possible explanation for this result is the magnitude of the associations: higher in the association between pacifier use duration and occurrence of AOB and lower in the association between breastfeeding duration and AOB. In other words, it is possible that the negative effect of prolonged use of a pacifier outweighs a possible benefit of breastfeeding on dental occlusion.

Two other Brazilian studies have reported similar results. Both observed negative association between occurrence of AOB and breastfeeding duration (< 12 months in one study [2] and < 9 months in the other [6]) in univariate analyses. However, again, in line with the findings of the present study, the associations lost significance when subjected to a multivariate analysis including pacifier use.

Conversely, in two other studies, one conducted in Brazil [15] and the other in France [1], the negative association between breastfeeding duration and occurrence of AOB remained significant even after adjustment for pacifier use. The main difference between the studies mentioned above and our investigation is that in those studies both breastfeeding duration and pacifier use duration were categorized.

A peculiar finding of our study was the fact that the association between longer breastfeeding duration and lower prevalence of AOB was significant when pacifier use duration was disregarded. For each month of breastfeeding, a reduction of 2% in the prevalence of AOB was estimated. It is possible that this estimate will vary depending on time of pacifier use, as the magnitude of the association between longer pacifier use duration and prevalence of AOB seems to be more robust than the association between longer breastfeeding duration and lower prevalence of AOB. For each month of pacifier use, the risk of AOB increased by 5%. Therefore, it seems reasonable to hypothesize that, as pacifier use duration increases, the protective effect of breastfeeding against development of AOB decreases. This may explain the loss of significance in the association between longer breastfeeding duration and lower prevalence of AOB when pacifier use duration was taken into consideration [13, 14, 17]. Moreover, longer pacifier use duration, in addition to causing orofacial myofunctional disorders, is associated with shorter breastfeeding duration, [18, 19] further increasing the risk for AOB.

The high prevalence of AOB found in this study (44.4%) can be explained, at least partly, by the high prevalence of pacifier sucking habits (42.6%) and bottle feeding (58.4%) among Brazilian children younger than 12 months, as pointed out in a survey conducted by the Brazilian Ministry of Health in 2009 [20]. It is also important to highlight that the municipality of Porto Alegre, where the study was conducted, showed the highest prevalence of pacifier use in that survey (59.5%) among all Brazilian state capitals [20].

The fact that the present study considered only one type of malocclusion, namely AOB, may be considered a limitation of the study, as well as not ruling out other causes of AOB during clinical examination. Moreover, a possible memory bias cannot be discarded, as data on breastfeeding duration and pacifier use between the age of 6 months and 3–5 years old were collected retrospectively. However, at least for breastfeeding duration, this type of bias is more relevant in the investigation of exclusive breastfeeding duration, [21] as mothers tend to remember total breastfeeding duration with relative accuracy. According to a study conducted in the United States, data on breastfeeding duration was slightly overestimated after 1–3.5 years of the outcome [22]. Another aspect to be taken into consideration is the different ages of the children upon assessment (ranging from 3 to 5 years). Nevertheless, we do not believe that this may have significantly influenced the results, especially because child age was included in the statistical analyses. Also, it is important to emphasize that malocclusions have multifactorial aetiology [5, 11]. This study considered only breastfeeding and pacifier use.

From a different standpoint, this study has the strength of being the first to express breastfeeding duration and pacifier use duration as continuous variables, allowing to quantify the risk of AOB for each additional month of breastfeeding and for each additional month of pacifier use. In addition, the results of this study contribute to reinforce the hypothesis that one of the mechanisms involved in the protective effect of breastfeeding against development of AOB is the ability that breastfeeding has to inhibit sucking habits (e.g. pacifier use) that are known to cause different malocclusion features, including AOB.

## Conclusions

This study showed that pacifier use duration might influence the association between longer breastfeeding duration and lower prevalence of AOB. The influence was more evident when pacifier use duration was considered, suggesting that new studies designed to investigate this association should include the actual duration of pacifier use in the analyses.

## Data Availability

The datasets used and/or analysed during the current study are available from the corresponding author on reasonable request.

## Abbreviations

AOB: Anterior Open Bite
SPSS: Statistical Package for Social Science
